# Estimation of the timing of *BAP1* mutation in uveal melanoma progression

**DOI:** 10.1038/s41598-021-88390-6

**Published:** 2021-04-26

**Authors:** Ogul E. Uner, Thonnie Rose O. See, Eszter Szalai, Hans E. Grossniklaus, Gustav Stålhammar

**Affiliations:** 1 Emory University School of Medicine, Atlanta, Georgia USA; 2 Departments of Ophthalmology and Pathology, Emory University School of Medicine, Atlanta, Georgia USA; 3 Department of Ophthalmology, University of Pécs Medical School, Pécs, Hungary; 4 St. Erik Eye Hospital, Stockholm, Sweden; 5 Department of Clinical Neuroscience, Karolinska Institutet, Stockholm, Sweden

**Keywords:** Eye cancer, Oncogenesis

## Abstract

Uveal melanoma is the most common primary intraocular malignancy. A vast majority of metastasizing tumors have mutations in the *BAP1* gene. Here, we investigate the spatiotemporal timing of these mutations. The size of 177 uveal melanomas and 8.3 million individual tumor cells was measured. *BAP1* sequencing results and BAP1 IHC were available and for 76 (43%) and 101 (57%) of these, respectively. Tumors with a *BAP1* mutation had significantly larger volume (2109 vs. 1552 mm^3^, *p* = 0.025). Similarly, tumor cells with loss of BAP1 protein expression had significantly larger volume (2657 vs. 1593 μm^3^, *p* = 0.027). Using observations of the time elapsed between mitoses, the *BAP1* mutation was calculated to occur when the primary tumor had a size of a few malignant cells to 6 mm^3^, 0.5 to 4.6 years after tumor initiation and at least 9 years before diagnosis. We conclude that *BAP1* mutations occur early in the growth of uveal melanoma, well before the average tumor is diagnosed. Its timing coincides with the seeding of micrometastases.

## Introduction

Uveal melanoma (UM) represents 3–5% of all melanomas and arises from the uvea, a pigmented region of the eye composed of the iris, choroid, and ciliary body^1^. Most tumors are located in the choroid (90%)^2^. It is the most common primary intraocular malignancy in adults, affecting approximately 5 individuals per million per year worldwide^2^. More than one third of patients suffer from metastatic spread, after which the median survival is less than 1 year^3,4^. Gene expression profiles are of great value in stratifying patient prognosis. Further, mutations in the BRCA1 associated protein-1 gene (*BAP1*), a tumor suppressor located on chromosome 3p, is mutated in 47% of all UM and a vast majority of metastasizing UM^5,6^. This mutation leads to a dedifferentiated stem-like phenotype that correlates strongly with poor prognosis^5,6^.

*BAP1* is one of many genes that undergo mutations to confer increased epithelial to mesenchymal transition of the tumor. In this mutation sequence, the *BAP1* mutation has been assumed to occur relatively late in tumor progression, preceded by oncogenic mutations in G-protein subunits including *GNA11* and *GNAQ* that are present in as high as 83–96% of UM^7^. It is thought that these subunit mutations are not sufficient for progression to metastatic disease. *BAP1* mutations are thought to appear after the *GNA11* or *GNAQ* mutations, correlating with a gratly increased risk for tumor progression^5^.

It is well-documented that larger UMs have a higher likelihood of harboring *BAP1* mutations, supporting prognostic implications^8,9^. However, there is also evidence that smaller UMs may harbor *BAP1* mutations and seed metastases^10^. The smallest UM of metastatic potential reported in the literature is 3.0 mm in diameter and 1.0 mm in thickness, corresponding to a volume of 4.7 mm^3^ in spherical cell models^11^. A study showed that of 61 choroidal melanomas with thickness ≤ 3 mm, 27% had monosomy 3^12,13^. Another study found 6 of 59 choroidal melanomas with thickness ≤ 3 mm and diameter ≤ 9 mm developed metastases after treatment, highlighting the metastatic potential of small UMs^14^. Additionally, there are also mathematical models that show seeding of liver micrometastases when the primary UM is less than 10.0 mm^3^ in size^12,13^. These findings support the thought that *BAP1* mutations can be present early in tumorigenesis and in small UMs.

Given the relationship between loss of BAP1 expression and poor prognosis, it is important to clarify the impact of tumor size and age on the likelihood of a *BAP1* mutation. To the best of our knowledge, no study has investigated these spatial and temporal characteristics in clinical samples. We therefore use mathematical modeling and previously published calculations of the tumor size at which the seeding of micrometastases start as a reference and comparison for our own model. Thus, we aim to make estimations of the origin and dynamic evolution of the *BAP1* mutated tumor cell clone in UM.

## Results

### Descriptive statistics

A total of 177 enucleated eyes with choroidal or ciliary body melanoma were included. *BAP1* sequencing results and BAP1 IHC were available and for 76 (43%) and 101 (57%) of these, respectively. Forty of the tumors with BAP1 IHC available were used as a validation cohort. The mean patient age at enucleation was 61 years (SD 15). One hundred and twenty-three patients (69%) had AJCC stage IIb or IIIa disease. The mean tumor volume was 913 mm^3^ (SD 865) and the mean number of cells measured in terms of size and BAP1 expression in each tumor was 208 403 (SD 165 587). Eighty-six patients were deceased before the end of follow-up. Median follow-up for the 91 survivors was 46 months (SD 78, Table 1).

**Table 1:** Characteristics of included patients and tumors.

***n***	177
Gender, n (%)	
Male	95 (54)
Female	82 (46)
Mean patient age at enucleation, years (SD)	61 (15)
Mean tumor thickness, mm (SD)	7.2 (4.0)
Mean tumor diameter, mm (SD)	13.8 (4.8)
Mean tumor volume, mm^**3**^ (SD)	913 (865)
**AJCC T-category, n (%)**	
1a	10 (6)
1b	0 (0)
1c	1 (1)
1b-d	0 (0)
2a	18 (10)
2b	4 (2)
2c-d	1 (1)
2d	0 (0)
3a	61 (34)
3b	9 (5)
3c	1 (1)
3d	0 (0)
4a	47 (27)
4b	17 (10)
4c	3 (2)
4d	3 (2)
4e	2 (1)
**AJCC stage, n (%)**	
I	10 (6)
IIa	19 (11)
IIb	65 (37)
IIIa	58 (33)
IIIb	20 (11)
IIIc	5 (3)
IV	0
BAP1 sequenced, n (%)	76 (49)
BAP1 IHC, n (%)	101 (51)
Mean cells measured/tumor, n (SD)	208 403 (165 587)
Median follow-up, months (SD)	46 (78)

Of 76 sequenced tumors, 26 (34%) had a *BAP1* mutation. Tumors with a *BAP1* mutation had significantly larger mean volume than those with wild-type *BAP1* (2109 vs. 1552 mm^3^, *p* = 0.025).

**Figure 1:**
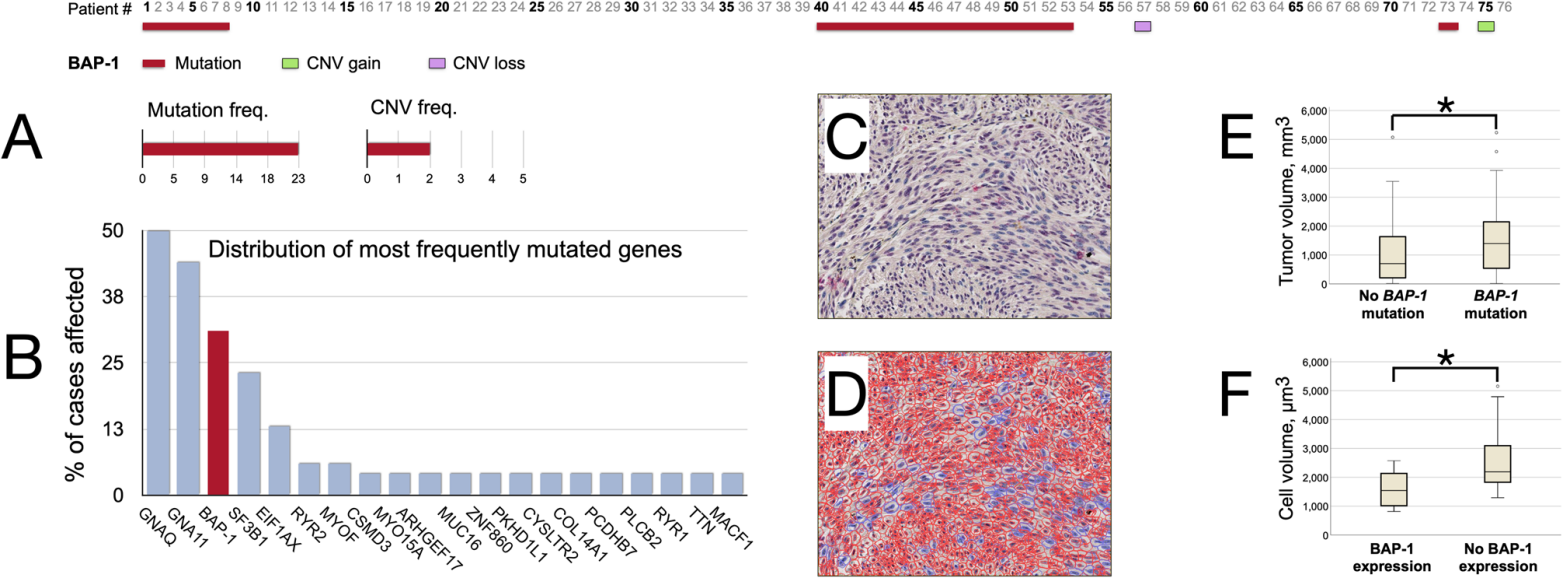
Results of whole exome sequencing and digital image analysis of BAP1 immunohistochemistry. (**A**) Distribution of *BAP1* mutations and copy number variations (CNV) across the 76 included patients from The Cancer Genome Atlas (TCGA, https://portal.gdc.cancer.gov/projects/TCGA-UVM). Twenty-two patients had a *BAP1* mutation, 1 patient had a *BAP1* CNV gain, and 1 patient had a *BAP1* CNV loss. (**B**) Distribution of the most frequently mutated genes as proportion of the cohort. (**C**) BAP1 immunohistochemistry. In this example, most tumor nuclei have retained their BAP1 expression (lilac) whereas some have lost their expression (blue). (**D**) Excerpt from digital image analysis of the same tumor section shown in B. Tumor cells with retained and lost BAP1 expression are indicated in red and blue, respectively. (**E**) Tumors with a *BAP1* mutation had significantly larger mean volume than those with wild-type *BAP1* (2109 vs. 1552 mm^3^, *p* = 0.025). (**F**) tumor cells with loss of BAP1 expression had significantly larger mean volume (2657 μm^3^, SD 1283) than those with retained expression (1593 μm^3^, SD 602, *p* = 0.027). Freq, Frequency. CNV, Copy number variation. * < 0.05.

Similarly, tumor cells with loss of BAP1 expression had significantly larger mean volume (2657 μm^3^, SD 1283) than those with retained expression (1593 μm^3^, SD 602, *p* = 0.027, Fig. 1). The mean volume of all tumor cells regardless of BAP1 expression was 2105 μm^3^ (SD 936).

### Cell doubling time

The mean number of tumor cells per tumor was 433 941 547 (SD 410 738 729), calculated by dividing each tumor’s volume with the mean tumor cell volume of 2105 μm^3^. The mean number of tumor cells with loss of BAP1 expression was 251 520 892 (SD 368 488 894). The mean number of cell doublings required to reach the number of tumor cells and number of tumor cells with BAP1 loss was 27.9 (SD 1.7, smallest tumor 24.2, largest tumor 30.7) and 25.8 (SD 3.2, smallest tumor 19.5, largest tumor 30.4), respectively. The difference of 2.1 tumor doublings indicate that the mutation occurred when the lesion consisted of only 4 malignant cells (n = 2^2.1^).

Median doubling times of 128 to 511 days in primary UM have been reported^15,16^. If each doubling took 128 days, the tumors in the present cohort consisted of 1 cell an average of 3571 days or 9.8 years before diagnosis (SD 0.6, min 8.5, max 10.8). Using the same doubling time for the cell population with loss of BAP1 expression, the first tumor cell with lost BAP1 expression appeared an average of 3302 days or 9.0 years before diagnosis (SD 1.1, min 6.8, max 10.7).

If the doubling time was 511 days instead, the first malignant cell appeared 39.1 years before diagnosis (SD 2.4, min 33.9, max 43.0), with the *BAP1* mutation occurring 3 years later at 36.1 years before diagnosis (SD 4.5, min 27.3, max 42.6).

### Curve fitting

We tested curves based on linear, logarithmic, inverse, quadratic, cubic, compound, power, S-shaped, logistic, growth and exponential functions. Goodness of fit to our data was tested in model summaries and ANOVA tables. In the first step, inverse, compound, power, power, S-shaped, growth, exponential and logistic functions were excluded as they could not be fitted to our data. All of the remaining functions (linear, logarithmic, quadratic and cubic) could be fitted (F-scores 3.2 to 9.8, R^2^ 0.10 to 0.15, *p* = 0.03 to 0.003, Table 2).

**Table 2:** Model summary of proportion of tumor cells with loss of BAP1 expression as a linear, logarithmic, quadratic and cubic function of tumor volume. *y*, proportion of tumor cells with loss of BAP1 expression. *x*, tumor volume in mm^3^.

Curve	Function	R^2^	F-score	*p*
Linear	*y* = 0.017*x* + 29.03	0.14	9.8	0.003
Logarithmic	*y* = −18.28 + 10.02ln(*x*)	0.10	6.4	0.01
Quadratic	*y* = 29.62 + 0.015*x* + 5.73E^−7^*x*^2^	0.14	4.8	0.01
Cubic	*y* = 32.24 + 0.003*x* + 1.07E^−5^*x*^2^ − 2.00E^−9^*x*^3^	0.15	3.2	0.03

Next, we used these functions to estimate the tumor volume at which the proportion of *BAP1* mutant cells is zero by setting the *y* value (proportion of tumor cells with loss of BAP1 expression) to 0. We thereby arrived at 0 to 6 mm^3^, depending on which of the functions were used for calculation. The latter corresponds to a lesion with a diameter of 5 mm and a thickness of 1.5 mm.

### Validation

We tested the linear, logarithmic, quadratic and cubic functions’ ability to solely based on tumor volume predict manual assessments of low versus high BAP1 expression (low < 33% BAP1 positive cells, high ≥ 33%) in a model validation cohort of 40 tumors. The included tumors had been independently graded by two ophthalmic pathologists’ ( H.E.G. and G.S.). True positive was defined as: Predicted to be positive (BAP1 ≥ 33%) by function and positive as graded by pathologist. True negative was defined as: Predicted to be negative (BAP1 < 33%) by function and negative as graded by pathologist.

The linear and quadratic functions predicted the classification of 65% of the cases, with a sensitivity and specificity of 31% and 88%, respectively and a Cohen’s Kappa of 0.21, indicating fair agreement.

The logarithmic function predicted the classification of 60% of the cases, with a sensitivity and specificity of 40% and 96%, respectively and a Cohen’s Kappa of 0.02, indicating slight agreement.

The cubic function predicted the classification of 65% of the cases, with a sensitivity and specificity of 69% and 88%, respectively and a Cohen’s Kappa of 0.57, indicating moderate agreement.

### Implications of doubling time and cell size in a large external cohort

We applied our measurements of tumor cells with lost and retained BAP1 protein expression to the mean tumor from a previously published cohort of 8 033 consecutive patients^4^. In this cohort, the mean tumor diameter and thickness at diagnosis of UM was 11.1 and 5.5 mm, respectively. Using the formula for calculation of tumor and cell volume described by Char et al., a UM with these measurements would have a volume of 348 mm^3^^12,16^. In a tumor with this volume, there would be approximately 165 320 665 tumor cells, if our mean tumor cell volume of 2105 μm^3^ μm^3^ was used.

As shown above, the volume of *BAP1* mutant cells can be described as a function of tumor volume. According to these functions, a tumor with a volume of 348 mm^3^ would have loss of BAP1 expression in 35 to 41% of its cells, or about 57 862 233 to 67 781 473 cells.

To reach 165 320 665 tumor cells, 27.3 cell doublings would be required. To reach the estimated number of cells with loss of BAP1 expression, 25.8 to 26.0 doublings would be required.

Consequently, the *BAP1* mutation occurred after 1.3 to 1.5 cell doublings, when the malignant clone consisted of only 3 malignant cells.

If each doubling took 128 days, a tumor with a volume of 348 mm^3^ consisted of 1 cell 3494 days, or approximately 9.5 years before diagnosis. Using the same doubling time for the cell population with loss of BAP1 expression, the first tumor cell with lost BAP1 expression appeared 3302 to 3328 days before diagnosis, when the tumor was 166 to 192 days or about 0.5 years old. If the doubling time was instead 511 days^16^, the tumor initiated 13 951 days or approximately 38.2 years before diagnosis and the first tumor cell with lost BAP1 expression appeared to 12 286 to 13 184 days or approximately 36.1 to 36.4 years before diagnosis. Consequently, with a doubling time of 511 days, the first tumor cell with lost BAP1 expression appeared when the tumor was 767 to 1665 days or 2.1 to 4.6 years old.

In summary, a tumor with a diameter of 11.1 mm and a thickness of 5.5 mm, which represents the average UM size at diagnosis, initiated 9.5 to 38.2 years before diagnosis, and its first cell with lost BAP1 expression appeared when the tumor was 0.5 to 4.6 years old, depending on the estimate of the proportion of tumor cells with loss of BAP1 expression in the average tumor and the mean time elapsed between mitoses.

### Survival

**Figure 2:**
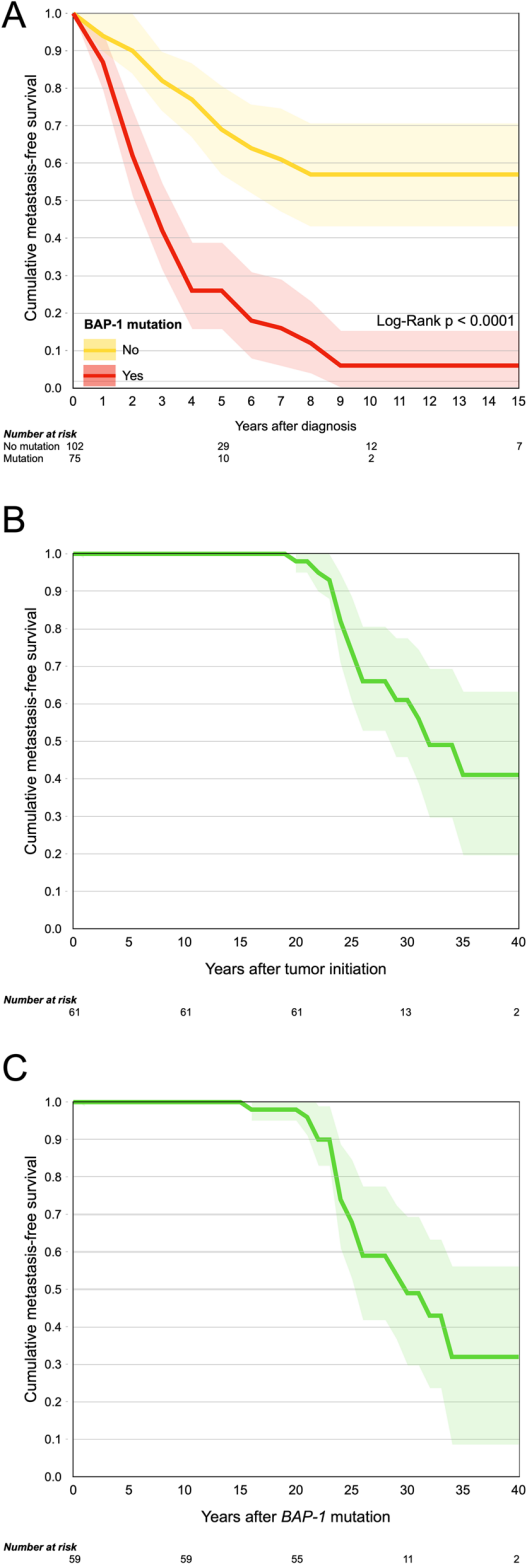
Cumulative metastasis-free survival proportions. (**A**) Survival after diagnosis for patients with a *BAP1* mutation or low immunohistochemical BAP1 expression (red) versus no BAP1 mutation or high immunohistochemical BAP1 expression (yellow, Log-Rank *p* < 0.0001). (**B**) Survival after tumor initiation, which was estimated to occur 22.3 years before diagnosis (SD 1.4). (**C**) Survival after *BAP1* mutation, which was estimated to occur 20.6 years before diagnosis (SD 2.5). Green areas represent 95% confidence intervals.

Median patient metastasis-free survival after diagnosis of tumors with a *BAP1* mutation was 2.4 years (standard error, SE 0.2, 95% confidence interval, CI 2.0 to 2.8), versus 16.0 years for tumors without a *BAP1* mutation (SE 7.6, 95% CI 1.2 to 30.9, Log-Rank *p* < 0.0001, Fig. 2A). For tumors with IHC stains, we used retained BAP1 expression in 33% of tumor cells as a cutoff, in accordance with previous publications^17–19^. Of 177 patients, 79 had metastases before the end of follow-up. Of these 79 metastases, 79 (100%) occurred within 9 years from diagnosis.

Lastly, we examined patient survival with the concepts metastasis-free survival after tumor initiation and metastasis-free survival after *BAP1* mutation. In contrast to using the day of diagnosis as starting point for survival analysis, we added the estimated time that had elapsed from tumor initiation and *BAP1* mutation *before* diagnosis. We assumed a cell doubling time of 292 days, which is the median of previous observations^16^.

Metastasis-free survival after tumor initiation was defined as the proportion of patients not having suffered symptomatic and/or radiologically detectable metastases at a specific time after the estimated appearance of the first tumor cell. With a doubling time of 292 days, the mean duration from tumor initiation to diagnosis was 22.3 years (SD 1.4). Median Kaplan–Meier metastasis-free survival after tumor initiation was 32.7 years (SE 3.2, 95% CI 26.4 to 39.1, Fig. 2B).

Metastasis-free survival after *BAP1* mutation was defined as the proportion of patients not having suffered symptomatic and/or radiologically detectable metastases at a specific time after the estimated appearance of the first tumor cell with loss of BAP1 expression. Two patients with BAP1 expression in 100% of their tumor cells were excluded from analysis, leaving 59 eligible patients. With a doubling time of 292 days, the mean duration from *BAP1* mutation to diagnosis was 20.6 years (SD 2.5). Median Kaplan–Meier metastasis-free survival after *BAP1* mutation was 31.0 years (SE 3.9, 95% CI 23.3 to 38.7, Fig. 2C).

## Discussion

**Figure 3:**
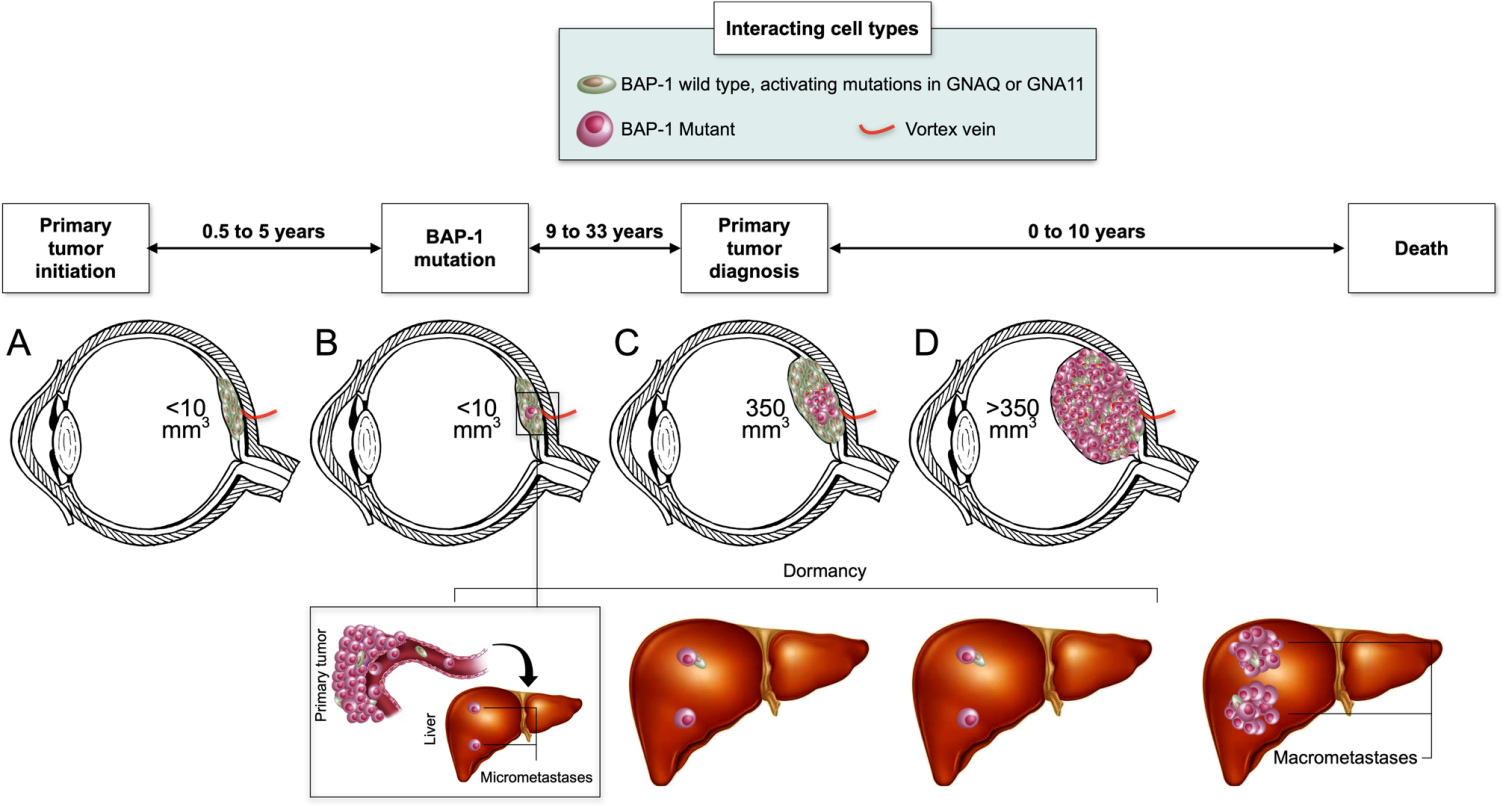
A model for the growth and dissemination of uveal melanoma. (**A**) In a tumor’s infancy, mutations in G-protein subunits including *GNA11* or *GNAQ* are accumulated. (**B**) In about half of uveal melanomas, subsequent mutations in *BAP1* occur after 0.5 to 5 years, at a tumor volume of < 10 mm^3^. At this point, micrometastases are seeded to the liver. (**C**) As the primary tumor keeps growing, the *BAP1* mutated clone has a survival advantage over *BAP1* wild type tumor cells, gradually replacing the latter. The average primary tumor is diagnosed at a volume of 350 mm^3^, 9.5 to 38 years after tumor initiation. Micrometastases may remain dormant and undetectable for years. (**D**) At a later stage, the primary tumor is composed of *BAP1* mutants only, with few exceptions. Eventually, liver micrometastases leave dormancy and start growing to radiologically detectable macrometastases. The entire process from primary tumor initiation to death from metastatic disease takes one to five decades. The minimum estimate is 9.5 years between tumor initiation and diagnosis, and 0 years from diagnosis to metastatic death for an overall course of 9.5 years. The maximum estimate is 38 years between tumor initiation and diagnosis, and 15 years from diagnosis to metastatic death for an overall course of 53 years. Later metastases are rare^3,22^. Note: The presence of micrometastases does not necessarily imply a worse prognosis. Subsequent events may be necessary for micrometastases to leave dormancy and start proliferating. Importantly, more than half of patients do not develop macrometastases at all^3,4,6,22,23^.

This study provides an estimate of the timing of the *BAP1* mutation in the growth of UM. Adding to previous publications on the metastatic process^20^ and to doubling times of the primary tumor and metastases, we propose a model for the growth and dissemination of UM (Fig. 3)^12,13,15,16^. Our calculations showed that the first *BAP1* mutant clone of an average UM appears when the tumor is 166 days to 1665 days old, within 2 cell doublings of the first UM clone. The timing of micrometastatic seeding has been estimated as 4.2 years after tumor initiation, which aligns with the calculated timing of the *BAP1* mutation^21^. Thus, we provide a mathematical explanation of the occurrence of micrometastases during the tumor’s infancy and underscore the importance of *BAP1* mutation in tumorigenesis.

It is important to note that our model applies to melanoma cells, which are malignant by definition. UM can occur de novo or through malignant transformation of a nevus. Studies have shown that choroidal nevi, similar to UM, can harbor G-protein coupled receptor (GPCR) mutations in *GNA11*, *GNAQ*, and *CYSLTR2*^24,25^. Our computational analyses assume that the initial cell is a malignant cell capable of losing the BAP1 gene, whether it formed de novo or growing in a nevus. To our knowledge, there are no *BAP1*-deficient choroidal nevi reported in the literature, so our model was able to focus solely on *BAP1* mutagenesis in the evolution of UM.

The early onset of the *BAP1* mutation is likely promoted by evolutionary pressure. Hypoxia and immune evasion may be two contributors. As the tumor grows, it will be at higher risk for hypoxia due to lower availability of nutrients. Studies of primary fibroblasts from individuals heterozygous for the germline *BAP1* mutation showed that these cells have increased anaerobic glycolysis and decreased mitochondrial respiration compared to fibroblasts from wild-type *BAP1* individuals^26,27^. This finding suggests that the *BAP1* mutated clone can be favored under hypoxic conditions and gradually make up a larger proportion of the tumor. Furthermore, if *BAP1* mutated cells are more stem-like, they also have a higher capability of immune evasion, thereby outliving the wild types. It has been documented that *BAP1* mutations can downregulate the expression of genes responsible for immune checkpoints and increase inflammation in the tumor microenvironment^28^. A study has shown that a liver metastasis that arose 29 years after treatment of a class 1B UM harbored clonally expanded plasma cells, suggesting the role of antibody-mediated immunity in slow tumor growth and immune evasion^28^. More recently, another study has shown the effect of aneuploidy, commonly seen with *BAP1* loss as monosomy 3, in immune suppression through activation of NF-kB and other pro-inflammatory signaling pathways^29^. Thus, both the hypoxic and stem-like properties would give *BAP1* mutants an evolutionary advantage over wild type cells.

Our study found significant heterogeneity in BAP1 expression within tumors, which requires exploration in the context of UM tumorigenesis. *BAP1* mutations tend to precede metastatic dissemination and they occur shortly after GPCR mutations in the UM evolutionary tree^28^. However, instances of monosomy 3 occurring after several UM mutations and cases in which metastases precede BAP1 loss have been reported^30–32^. A recent study identified 2 cases where primary UM homozygous for the *BAP1* mutation gave rise to *BAP1* heterozygous metastases^30^. However, metastases in both cases were negative for the BAP1 protein. This could be explained by *BAP1* epigenetic silencing or the escape of the mutation from detection, as the authors suggest. That study utilized targeted resequencing with 423-fold coverage and without methylomic analysis, so it may have not detected smaller mutations in metastases. However, if this is replicated in additional studies, it would suggest that *BAP1* loss can be clonal, supporting the observation of intratumor *BAP1* heterogeneity in our study. Another investigation used digital PCR analysis to show that the order monosomy 3 shows heterogeneity with respect to other mutagenic events like gain in chromosome 8q^31^. Most recently, we found that there is significant intratumor heterogeneity of BAP1 expression, but that this has limited prognostic importance^33^. Robust applications of genomic analysis, such as digital PCR offering over 5000-fold coverage, are required to continue investigating the dynamic mutagenesis of *BAP1* and tumor heterogeneity in UM.

It is important to note the role of tumor architecture and vascular framework, apart from the *BAP1* mutation, in the promotion of metastasis in UM. Our study suggests that a *BAP1* mutation occurs early in tumorigenesis, possibly when the tumor consists of a few malignant cells. However, this does not imply immediate initiation of metastases. Once a cell loses BAP1 expression, even after the GPCR mutation that drives proliferation, it needs to extravasate into vascular channels of the choroid. The necessary tumor architecture at this stage needs to be established and consists of small vascular channels in the primary tumor. It has been shown that tumors with high-grade vascularity patterns are significantly more likely to metastasize than tumors that do not, with mean vascular density positively correlating with the number of metastases, even in small UMs^34^. In another study, we showed that low *BAP1* expression correlates with areas with areas of vasculogenic mimicry, consisting of de novo formations of tubules without endothelial lining that connect the UM to mature vasculature^35^. Thus, the requirements of the tumor microenvironment could explain the delay between the origination of the *BAP1* mutant clone, the *BAP1-*GPCR double mutant clone, and micrometastatic spread.

The strengths of our study include its relatively large patient sample and use of our model in several different cohorts. By formulating this mathematical model, this study provides an early snapshot of *BAP1* mutagenesis and suggests micrometastases can occur as early as after the first few mitoses of primary UM. One limitation of this study is its theoretical nature. We assumed cells were ellipsoid in shape, which is a frequently used assumption but not necessarily accurate for each individual tumor cell. Our model solely incorporated the *BAP1* mutation. Several other mutations, such as *EIF1AX* and *SF3B1*, less often promote UM metastasis. Thus, a combined model may have increased the accuracy of our results. Since UM doubling times were calculated under separate postulations in the literature, we were only able to present our estimates in intervals and assume these values were constant. This is likely not the case, as we found BAP1 expression is a function of tumor volume. Our model did not take dynamic changes in the tumor environment during growth into account, including other mutations, chromosomal status, dormancy intervals and changes in tumor doubling time. Furthermore, from the observation that larger tumors harbor a greater proportion of *BAP1* mutant cells follows that *BAP1* mutated clone should have either a faster growth rate or a slower death rate. This assumption has not been used to modify calculations in the current study, and future studies may clarify this assumption based on observations rather than estimations. Some authors may question our findings that UMs contains a ratio of *BAP1* mutant-to-wild type cells, arguing that UM is more often composed exclusively of mutants or wild-types. In our study, we have alternately used loss of immunohistochemical expression of BAP1 in tumor cell nuclei and *BAP1* mutations detected by DNA sequencing as a marker for the same genetic event, using methods replicated in our previous studies^17–19^. It is also important to note that the presence of micrometastases is common regardless of BAP1 status^23,36^. Events that trigger micrometastases to leave dormancy and start proliferating in some patients may be even more important in terms of survival, considering that more than half of patients with UM do not develop metastases at all.

In conclusion, the *BAP1* mutation occurs early in the growth of UM, well before the average tumor is diagnosed and the timing coincides with previous calculations of the tumor size at which seeding of micrometastases start. At the time of primary tumor diagnosis, the primary tumor, the *BAP1* mutation and the liver micrometastases are one to several decades old. Considering an average patient age of about 60 years at diagnosis, these crucial tumor events occurred at an age of 20 to 50 years. Further studies of *BAP1* mutagenesis and on factors that promote micrometastasis dormancy and reduce the risk for a switch to proliferation could improve the prospects for development of effective therapies and improved survival.

## Methods

### Patients and samples

The protocol for data collection and analysis of specimens from the L.F. Montgomery Ophthalmic Pathology Laboratory of Emory Eye Center, Atlanta, USA, was approved by the Emory University Institutional Review Board (reference 00,028,367) and from the Ophthalmic Pathology Laboratory of St. Erik Eye Hospital in Stockholm, Sweden by the Swedish Ethical Review Authority (reference 2020–02,835). The study adhered to the US Health Insurance Portability and Accountability Act and to the tenets of the Declaration of Helsinki. No protected health information was collected or handled in this project. All used tumor and patient data had been generated in previously published projects and was anonymized at the point of our analysis in this project.

The 177 included patients were collected from 3 different cohorts:

The first cohort (*n* = 61) consists of paraffin embedded and formalin-fixed eyes that were enucleated at St. Erik Eye Hospital and Emory Eye Center between years 1975 and 2017. They were included based on a set of inclusion criteria [histologically proven melanoma in choroid and/or ciliary body, patient deceased (requirement for waiver of informed consent by the Swedish Ethical Review Authority), availability of gene expression classification, sufficient formalin-fixed paraffin-embedded (FFPE) tissue for immunohistochemical staining and proper representation of tumor histopathology, availability of clinicopathological data including primary tumor largest basal diameter (LBD) and tumor thickness] and exclusion criteria (extensive tumor necrosis, hemorrhage or inflammation, abundant tumor pigmentation affecting visual examination of immunohistochemical protein expression and suboptimal staining results as determined by positive and negative internal and external controls). This cohort was published in 2019 and 2020^10,18,19,37^.

The second cohort (*n* = 76) was collected from The Cancer Genome Atlas (TCGA), the National Cancer Institute, National Institutes of Health, USA. In supplemental information to the publication by Robertson et al. anonymized patient and tumor data including results from whole exome sequencing have been made available^38^. This data was downloaded to calculate the difference in tumor volume between tumors with and without a *BAP1* mutation. The cohort originally consists of 80 patients, but 4 were excluded since they did not have complete tumor size data. This cohort was published in 2017^38^.

The third cohort (*n* = 40) was collected from the Emory Eye Center for validation of findings. The included tumors had been independently graded for the level of BAP1 expression by two authors (E.S. and H.E.G.). This cohort was published in 2018^17^.

### Immunohistochemical staining and digital image analysis

Formalin fixed and paraffin-embedded (FFPE) tumor sections had been stained with monoclonal antibodies against BAP1 with a red chromogen (1:40, Santa Cruz Biotechnology, Dallas, Texas, USA). The Leica Bond-III automated system (Leica, Wetzlar, Germany) and digitally scanned to the .ndpi file format at × 400, using the Nano Zoomer 2.0 HT scan (Hamamatsu Photonics K.K., Hamamatsu, Japan). The QuPath Bioimage analysis software (v0.2.3) run on an Apple computer (Apple Inc., Cupertino, CA), had been used for digital image analysis^39^. Each full tumor section was then analyzed for the number of tumor cells, number of cells with loss of BAP1 expression and retained expression, respectively, and size measurements for each individual cell (maximum and minimum caliper). Cell measurements were obtained from the 61 tumors in the first cohort and were validated in the second and third cohorts. A workflow for morphometric analysis was established using the QuPath software. The staining, scanning, digital image analysis, and cell measurement steps used for the included cohorts have been described previously^10,18,19,33^.

### Estimation of tumor and cell volume

The volume of tumors was estimated assuming a semi ellipsoid shape^12,16^:

$$ {\text{Volume of tumor}} = \frac{{\uppi }}{6} \times t \times lbd^{2} $$

where *t* is the tumor thickness and *lbd* is the largest basal diameter.

In calculations of the volume of tumor cells we assumed a prolate ellipsoid shape:

$$ {\text{Volume of cell}} = \frac{4}{3} \times {\uppi } \times ab^{2} $$

where *a* and *b* are the long and short calipers of the cell, respectively.

The number of cell doublings required to reach the number of cells in a tumor (x) was calculated as:

$$ 2^{x} { } = {\text{ number of tumor cells}} $$

### Model computation and statistical analysis

Differences with a *p* < 0.05 were considered significant, all *p* values being two-sided. Continuous variables including cell and tumor sizes did not deviate significantly from normal distribution, when evaluated by the Shapiro–Wilk test (*p* = 0.64). We therefore used the Student’s T-test for comparison of these variables. The ANOVA F-test and coefficient of determination (R^2^) was used to optimize the computational model and goodness of fit of S, compound, logistic, growth, exponential, linear, logarithmic, inverse, quadratic and cubic curves of the proportion of *BAP1* mutated cells as a function of tumor size. Under the assumption that the cells are ellipsoid in shape, the volume formula for a rotating ellipsoid was used to calculate cell volumes. We used binary logistic regression without x-centering of the tumor volume variable to predict the probability of a BAP1 mutation as a function of the tumor volume. X-centering was defined as inclusion of the constant in the regression model. The range of doubling times of a given tumor was assumed to be 154–511 days^12,13^. Follow-up was defined as the time in months from uveal melanoma diagnosis to the last occasion metastasis-free patients were known to be alive. All statistical analyses were performed using IBM statistics version 26 (Armonk, NY, USA).
