# Author Correction: Estimation of the timing of *BAP1* mutation in uveal melanoma progression

**DOI:** 10.1038/s41598-021-96113-0

**Published:** 2021-08-17

**Authors:** Ogul E. Uner, Thonnie Rose O. See, Eszter Szalai, Hans E. Grossniklaus, Gustav Stålhammar

**Affiliations:** 1 Emory University School of Medicine, Atlanta, Georgia USA; 2 Departments of Ophthalmology and Pathology, Emory University School of Medicine, Atlanta, Georgia USA; 3 Department of Ophthalmology, University of Pécs Medical School, Pécs, Hungary; 4 St. Erik Eye Hospital, Stockholm, Sweden; 5 Department of Clinical Neuroscience, Karolinska Institutet, Stockholm, Sweden

Correction to: *Scientific Reports* https://doi.org/10.1038/s41598-021-88390-6, published online 26 April 2021

The original version of this Article contained an error in the legend of Figure 1.

“F) Tumor cells with loss of BAP1 expression had significantly larger mean volume (801 μm^3^, SD 223) than those with retained expression (524 μm^3^, SD 352, *p* = 0.042).”

now reads:

“**(F)** tumor cells with loss of BAP1 expression had significantly larger mean volume (2657 μm^3^, SD 1283) than those with retained expression (1593 μm^3^, SD 602, *p* = 0.027).”

The original Article has been corrected.

